# A single tree model to consistently simulate cooling, shading, and pollution uptake of urban trees

**DOI:** 10.1007/s00484-020-02030-8

**Published:** 2020-10-18

**Authors:** Rocco Pace, Francesco De Fino, Mohammad A. Rahman, Stephan Pauleit, David J. Nowak, Rüdiger Grote

**Affiliations:** 1 Institute of Meteorology and Climate Research–Atmospheric Environmental Research (IMK-IFU), Karlsruhe Institute of Technology (KIT), Garmisch-Partenkirchen, Germany; 2 Institute of Research on Terrestrial Ecosystems (IRET), National Research Council (CNR), Porano, Italy; 3 Department of Informatics, Bioengineering, Robotics and Systems Engineering (DIBRIS), University of Genoa, Genoa, Italy; 4 Chair for Strategic Landscape Planning and Management, School of Life Sciences Weihenstephan, Technische Universität München, Munich, Germany; 5 USDA Forest Service, Northern Research Station, Syracuse, NY USA

**Keywords:** Microclimate model, Transpiration, Shading, Stomatal conductance, Soil water availability, Urban green spaces

## Abstract

Extremely high temperatures, which negatively affect the human health and plant performances, are becoming more frequent in cities. Urban green infrastructure, particularly trees, can mitigate this issue through cooling due to transpiration, and shading. Temperature regulation by trees depends on feedbacks among the climate, water supply, and plant physiology. However, in contrast to forest or general ecosystem models, most current urban tree models still lack basic processes, such as the consideration of soil water limitation, or have not been evaluated sufficiently. In this study, we present a new model that couples the soil water balance with energy calculations to assess the physiological responses and microclimate effects of a common urban street-tree species (*Tilia cordata* Mill.) on temperature regulation. We contrast two urban sites in Munich, Germany, with different degree of surface sealing at which microclimate and transpiration had been measured. Simulations indicate that differences in wind speed and soil water supply can be made responsible for the differences in transpiration. Nevertheless, the calculation of the overall energy balance showed that the shading effect, which depends on the leaf area index and canopy cover, contributes the most to the temperature reduction at midday. Finally, we demonstrate that the consideration of soil water availability for stomatal conductance has realistic impacts on the calculation of gaseous pollutant uptake (e.g., ozone). In conclusion, the presented model has demonstrated its ability to quantify two major ecosystem services (temperature mitigation and air pollution removal) consistently in dependence on meteorological and site conditions.

## Introduction

The increasing occurrence of heatwaves due to global warming (Perkins et al. 2012; Baldwin et al. 2019) presents a serious threat to human health (Watts et al. 2019). For example, the 2003 heatwave in Europe caused more than 70,000 deaths (Robine et al. 2008) and the heatwave-related premature mortality is expected to increase at the global scale (Guo et al. 2018). Extreme high temperatures are prevalent in cities, where the high percentage of sealed surfaces contributes to the so-called heat island effect (Oke 2002; Wilby 2003). Therefore, measures to mitigate the temperature stress are intensively discussed. The implementation of green infrastructure is arguably the most prominent suggestion to initiate this (Norton et al. 2015) and indeed, the associated cooling effect has been evaluated in many observational studies (Bowler et al. 2010; Rahman et al. 2020a). In particular, it is suggested to increase the number of urban trees in order to enhance the cooling effect (Zölch et al. 2016).

Trees reduce the air temperature in two ways: by preventing solar radiation from heating up surfaces below the canopy (shading) and by converting energy to latent heat flux through the transpiration of water released through the stomata of leaves (in the following termed cooling; Rahman et al. 2020a). However, these effects strongly depend on the tree dimensions and physiology and thus the climatic conditions (Rahman et al. 2018). In particular, low relative humidity (or high vapor pressure deficit) triggered by high temperature reflects a high transpiration demand. However, the actual transpiration is limited by the soil water availability. Insufficient water supply causes stomata closure and decreases the water uptake and thus the cooling effect of transpiration (Rötzer et al. 2019). Other meteorological factors that are positively related to evaporation are solar radiation, because stomata tend to open if the radiation is high, and the wind speed, which reduces the boundary layer thickness and thus the resistance to water transport from the canopy (Kramer 1983).

Several approaches have been considered to simulate the effect of vegetation on the micrometeorology in urban environments. The most prominent ones are computational fluid dynamics models that are built on fundamental fluid mechanics and thermodynamics laws and are used to calculate the interactions between trees and their surroundings (Buccolieri et al. 2018). Urban canopy models based on which the energy budget is determined with a simplified consideration of the flows represent a simpler approach (e.g., Lee and Park 2008; Krayenhoff et al. 2014; Zeng and Gao 2017). Recently, the use of large-eddy simulation models has been suggested for the estimation of the mitigating effect of vegetation on urban heat, although they currently do not fully consider the impact of evaporation (Li and Wang 2018). However, all these model types are computational demanding, difficult to parameterize, and very sensitive to the initial boundary conditions. Therefore, it is difficult to consider different species or individual properties, which diminishes their value as decision support tools for urban planning.

Urban forest-specific models represent a different group of models, most prominent in urban ecosystem research (Lin et al. 2019). These models focus less on the interactions between vegetation and the environment but more on the physical and physiological properties and processes of plants, that is, trees. A prominent example is the i-Tree model that can be used to estimate a broad range of ecosystem functions such as the air pollution removal, carbon sequestration, and building energy conservation (Nowak et al. 2008; Endreny et al. 2017). Among these services, energy estimates are related to the cooling function of trees (Scholz et al. 2018), but are mainly related to tree shade (Mcpherson and Simpson 1999). Because cooling is arguably the most important ecosystem service regarding the mitigation of future climate change effects in cities, this function must be calculated explicitly, that is, the effects of stomatal conductance and water limitation must be clarified. The combination of shading and leaf area or crown coverage or cooling and evapotranspiration has only been attempted in a few studies (Yang et al. 2013; Rötzer et al. 2019).

If evapotranspiration is calculated based on the stomatal conductance, another ecosystem service, that is, the gaseous pollutant uptake, can be physically considered. In contrast to particle deposition, gaseous pollutants, such as ozone, are primarily taken up through the stomata (Hosker and Lindberg 1982). When stomata are open, O_3_ diffuses into the intercellular space from the atmosphere and is almost immediately destroyed by antioxidant reactions with membrane lipids, moisture, and cell organelles in the apoplast (Tiwari et al. 2016). Stomatal O_3_ uptake depends strongly on stomatal conductance and therefore, uptake rates differ depending on the assimilation conditions or water supply of the plants (Fitzky et al. 2019). Among the various models that calculate stomatal uptake, few consider the soil water limitation, for example, the ozone deposition DO3SE model (Emberson et al. 2000; Büker et al. 2012). This type of model is primarily used to evaluate the effect on plants rather than on the environment because it was designed for the evaluation of trade-off between drought stress and ozone stress (Sicard et al. 2016). However, until very recently, we were not aware of any model that jointly considers the effects of limited water supply on both the cooling function and air pollutant removal of trees.

In this study, we introduce a single tree model coupled to a one-dimensional soil water model to consider the effect of drought stress on stomatal conductance and thus on cooling as well as air pollution removal functions. The objective is to demonstrate that the model works realistically and could be applied as a decision-support tool based on general available data. Therefore, we calculated the temperature mitigation (cooling and shading) of a common street-tree species (*Tilia cordata*) and evaluated these data with measurements from the literature. In addition, we use the evaluated model to demonstrate the potential impact of soil water conditions on the uptake of ozone.

## Methods

### Study area and local data

The study areas are located in Munich (Germany) and have different topographical features: Bordeaux Platz is an open green square and Pariser Platz is a circular paved square. Five trees of *T. cordata* with different morphological characteristics have been measured in each location (Table 1). Canopy cover was calculated from crown radii which were measured in eight inter cardinal directions and LAI was derived from hemispherical photographs captured during the fully leafed phase (June–August) as described in Rahman et al. (2017a). Trees at Bordeaux Platz were typically smaller than those at Pariser Platz.

**Table 1:** Average morphological characteristics of trees at the two study sites (*DBH* diameter at 1.3 m height, *LAI* leaf area index)

Sites	DBH (cm)	Height (m)	Canopy cover (m^2^)	LAI
Bordeaux Platz	29.18 ± 0.52	15.12 ± 0.21	67.12 ± 3.37	2.41 ± 0.19
Pariser Platz	44.68 ± 1.27	16.78 ± 0.29	81.7 ± 3.97	2.54 ± 0.18

Sap flux density measurements carried out from July 29 to August 31, 2015, were used to estimate the tree transpiration. Summer 2015 was an exceptionally hot and dry summer and represents a crucial time of year for evaluating the effect on stomatal conductance. Tree core samples were used to estimate tree age and determine sapwood area of trees which is the basis for determining sap flow rate. In order to facilitate the direct comparison of the trees, the water use was then scaled for each tree to a DBH and sapwood area at an age of 40 years.

The soil moisture potential was measured throughout the soil profile to a depth of 30 cm and local data, including the air temperature, pressure, relative humidity, and wind speed, were measured at the two study sites. Furthermore, the global radiation was measured at Bordeaux Platz; all data were continuously recorded at resolution of 15 min. Precipitation data were derived from the *Theresienstrasse* weather station in Munich. Missing values on August 5th are due to vandalism-related measurement problems. For more details on the methodology, please see Rahman et al. (2017a).

### Model calculations

#### Transpiration, stomatal conductance, and O_3_ deposition velocity

The transpiration flux (*T*_f_, g m^−2^ h^−1^) was calculated using the i-Tree Eco model methodology (Hirabayashi et al. 2015). The amount of water evaporating through stomata is controlled by the leaf and boundary layer resistances (Kramer 1983):

1 $${T}_{\mathrm{f}}=\frac{C_{\mathrm{leaf}}-{C}_{\mathrm{a}\mathrm{ir}}\ }{\frac{1}{\mathrm{gs}}+{R}_{\mathrm{a}}}\bullet \frac{3600}{\mathrm{LAI}},$$

where *C*_leaf_ is the water vapor concentration of evaporating surfaces within the leaf (g m^−3^), *C*_air_ is the water vapor concentration in the air (g m^−3^), 1/gs is the stomatal resistance (s m^−1^, gs = stomatal conductance), *R*_a_ is the aerodynamic resistance (s m^−1^), and LAI is the leaf area index.

The parameters *C*_leaf_ and *C*_air_ can be calculated as follows (Monteith and Unsworth 2013):

2a $${C}_{\mathrm{leaf}}=\frac{M_{\mathrm{w}}\ {e}_{\mathrm{s}}}{R\ T}$$

2b $${C}_{\mathrm{air}}=\frac{M_{\mathrm{w}}\ e}{R\ T},$$

where *M*_W_ is the molecular weight of water (18 g mol^−1^), *R* is the universal gas constant (8.314 J mol^−1^ K^−1^), *e*_s_ is the saturation vapor pressure (kPa), *e* is the vapor pressure (kPa), and *T* is the temperature (K).

The stomatal conductance of each layer of the canopy can be calculated based on the methods explained in Farquhar et al. (1980), Baldocchi (1994), and Harley et al. (1992):

3 $$\mathrm{gs}=\frac{m\ A\ \mathrm{rh}}{C_{\mathrm{s}}}+\mathrm{gm},$$

where *m* is the Ball–Berry coefficient, *A* is the photosynthetic carbon flux into the leaf, rh is the relative humidity, *C*_s_ is the CO_2_ concentration at the leaf surface, and gm is the minimum stomatal conductance (0.02 mol m^−2^ s^−1^) when the stomata are closed (*A* = 0) assuming a cuticular resistance of 0. The terms *m* and gm are the slope and intercept of the relationship between assimilation and stomatal conductance, obtained by linear regression of gas exchange measurements.

The aerodynamic resistance (*R*_a_) is calculated as follows:

4 $${R}_{\mathrm{a}}=\frac{u(z)}{{u_{*}}^2},$$

where *u*(*z*) is the mean wind speed at the height of the weather station *z* (m s^−1^) and u_*_ is the friction velocity (m s^−1^).

The O_3_ deposition velocity (vd) can be calculated as the inverse of the sum of the aerodynamic (*R*_a_), quasi-laminar boundary layer (*R*_b_), and canopy (*R*_c_) resistances, expressed in s m^−1^ (Baldocchi et al. 1987):

5 $$\mathrm{vd}=\frac{1}{R_{\mathrm{a}}+{R}_{\mathrm{b}}+{R}_{\mathrm{c}}}$$

The canopy resistance (Rc) is calculated as:

6 $$\frac{1}{\mathrm{Rc}}=\frac{1}{r_{\mathrm{s}}+{r}_{\mathrm{m}}}+\frac{1}{r_{\mathrm{s}\mathrm{oil}}}+\frac{1}{r_{\mathrm{t}}},$$

where *r*_s_ is the stomatal resistance (s m^−1^), *r*_m_ is the mesophyll resistance (s m^−1^), *r*_soil_ is the soil resistance (2941 s m^−1^), and *r*_t_ is the cuticular resistance (s m^−1^).

The quasi-laminar boundary layer (*R*_b_) is calculated as:

7 $${R}_{\mathrm{b}}=2{\left(\mathrm{Sc}\right)}^{\frac{2}{3}}\ {\left(\Pr \right)}^{-\frac{2}{3}}\ {\left({ku}_{*}\right)}^{-1},$$

where Sc is the Schmidt number (1), Pr is the Prandtl number (0.72), *k* is the von Karman constant (0.41), and u_*_ is the friction velocity (m s^−1^).

#### Water balance model

The water balance is based on the DeNitrification and DeComposition (DNDC) model (Li et al. 1992) modified for urban conditions and short-term calculations. The following water fluxes are considered:

8 $$P=T+I+E+R+S,$$

where *P* is precipitation, *T* is transpiration, *I* is interception, *E* is the evaporation, *R* is runoff, and *S* is seepage (percolation below the last considered soil layer). The model determines daily potential evapotranspiration from daily temperature based on a modified Thornthwaite equation (Thornthwaite and Mather 1957) and considering the dependency on the latitude (Camargo et al. 1999; Pereira and Pruitt 2004). The potential demand for hourly evaporation was determined by dividing the daily evaporation by 24, which has shown to work well in various applications where this model has been applied to describe ecosystem processes (Holst et al. 2010; Magh et al. 2019). The interception is assumed to be linearly related to the LAI and retained water evaporates from leaves according to the evaporation demand. Water drawn from the soil by evaporation and transpiration can be calculated as the minimum of either the remaining potential evapotranspiration or water demand, which in turn depends on photosynthesis and the species-specific water-use efficiency (3 μmol mmol^−1^; Gillner et al. 2015). The soil evaporation was determined from the residual evaporation demand and soil water available below a predefined depth (0.3 m). The water movement within the soil depends on the difference between the relative water contents of the three adjacent soil layers and is regulated by the soil hydraulic conductivity. For Pariser Platz, we assumed a runoff of 40% due to the impervious surface and lower soil depth (0.1, 0.2, and 0.4 m for the three layers, respectively, compared with 0.2, 0.3, and 0.5 m, respectively, at Bordeaux Platz). A drought index (DI) was defined to limit the stomatal conductance and reduce the Ball–Berry constant (*m*) from 10 to 3 according to the soil water availability:

9 $$\mathrm{DI}=\frac{\left(\mathrm{water}\ \mathrm{content}-\mathrm{wilting}\ \mathrm{point}\right)}{\left(\mathrm{field}\ \mathrm{capacity}-\mathrm{wilting}\ \mathrm{point}\right)},$$

where:

• *if* DI ≤ 0.3 → *m* = 3.

• 0.3 < DI < 0.5 → *m* = 3 + 35 × (DI − 0.3)

• DI ≥ 0.5 → *m* = 10

#### Energy balance model

Energy reduction based on cooling and shading was evaluated at midday (12:00–15:00, CET). The average hourly transpiration *T* (ml h^−1^ m^−2^) was converted into energy loss (W m^−2^) by multiplication with the latent heat of vaporization *L*_V_, which is 2450 J kg^−1^ and division by 3600 s:

10 $${E}_{\mathrm{cooling}}=\frac{T\times {L}_{\mathrm{v}}}{3600}$$

The net rate of heat flow to and from a pavement surface (*q*_net_) can be calculated as follows (Solaimanian and Kennedy 1993):

11 $${\mathrm{q}}_{\mathrm{net}}={q}_{\mathrm{a}}+{q}_{\mathrm{s}}-{q}_{\mathrm{c}}-{q}_{\mathrm{k}}-{q}_{\mathrm{r}},$$

where *q*_a_ is the absorbed energy from direct solar (shortwave) radiation, *q*_s_ is the atmospheric radiation absorbed by the pavement surface, *q*_c_ is the convection energy, *q*_k_ is the conduction energy, and *q*_r_ is the surface emission.

12 $${q}_{\mathrm{a}}=\left(1-a\right)\times R,$$

where *a* is the albedo (0.3) and *R* is the direct radiation (determined from the global radiation as described in Spitters et al. 1986).

13 $${q}_{\mathrm{s}}={\varepsilon}_{\mathrm{a}}\sigma\ {T_{\mathrm{a}\mathrm{ir}}}^4$$

${\varepsilon}_{\mathrm{a}}=0.77-0.28\times {10}^{\left(-{V}_{\mathrm{p}}\times 0.074\right)}$ (Geiger 1959), *V*_p_ = vapor pressure (mmHg), *σ* = Stefan–Boltzmann constant (= 5.68 × 10^−8^ W m^−2^ K^−4^), *T*_air_ = air temperature (K)

14 $${q}_{\mathrm{c}}={h}_{\mathrm{c}}\left({T}_{\mathrm{s}}-{T}_{\mathrm{air}}\right)$$

*h*_c_ = surface coefficient of heat transfer =698.24[0.00144 *T*_m_^0.3^*U*^0.7^ + 0.00097(*T*_s_ − *T*_air_)^0.3^], *T*_s_ = surface temperature (K), *T*_m_ = average of the surface and air temperature (K), *U* = average daily wind speed (m s^−1^)

15 $${q}_{\mathrm{k}}=-k\frac{T_d-{T}_{\mathrm{s}}}{d}$$

*k* = thermal conductivity (1.65 W m^−1^ K^−1^), *d* = depth (2 m), *T*_*d*_ = temperature at depth *d* (8 °C)

16 $${q}_{\mathrm{r}}={\varepsilon}_{\mathrm{b}}\sigma\ {T_{\mathrm{s}}}^4$$

with

*ε*_b_= $$1.24\times {\left(10\times {V}_{\mathrm{p}}/{T}_{\mathrm{air}}\right)}^{\frac{1}{7}}$$ (Brutsaert 1982).

The reduction of the direct radiation through the tree crown was calculated using a modified Beer–Lambert law considering a uniform leaf arrangement in the canopy:

17 $${R}_{\mathrm{in}}=R\times {e}^{-k\times \mathrm{LAI}},$$

where *R*_in_ is the irradiance under the tree canopy (W m^−2^) and *k* is the extinction coefficient (0.7 for deciduous forest).

The equilibrium temperature at the pavement surface is calculated by setting the net rate of heat flow (*q*_net_) to 0:

18 $${q}_{\mathrm{a}}+{q}_{\mathrm{s}}-{q}_{\mathrm{c}}-{q}_{\mathrm{k}}-{q}_{\mathrm{r}}=0$$

The equation obtained considers surface temperature, air temperature, and temperature at a depth where heat flow can be assumed zero. The surface temperature can be thus calculated by knowing *T*_air_ from measurements and assuming a constant annual average temperature *T*_*d*_ (8.0 °C) at 2-m depth.

The energy reduction by shading (*E*_shading_; W m^−2^) was calculated as the absolute difference between the energy balance outside and inside the tree canopy:

19 $${E}_{\mathrm{s}\mathrm{hading}}=\mid {q}_{\mathrm{a},\mathrm{out}}-{q}_{\mathrm{a},\mathrm{in}\mid }+\left|{q}_{\mathrm{s},\mathrm{out}}-{q}_{\mathrm{s},\mathrm{in}}\right|+\left|{q}_{\mathrm{c},\mathrm{out}}-{q}_{\mathrm{c},\mathrm{in}}\right|+\left|{q}_{\mathrm{k},\mathrm{out}}-{q}_{\mathrm{k},\mathrm{in}}\right|+\left|{q}_{\mathrm{r},\mathrm{out}}-{q}_{\mathrm{r},\mathrm{in}}\right|$$

The total energy reduction *E* (W m^−2^) was calculated as the sum of *E*_shading_ (W m^−2^) and *E*_cooling_ (W m^−2^).

## Results

**Fig. 1:**
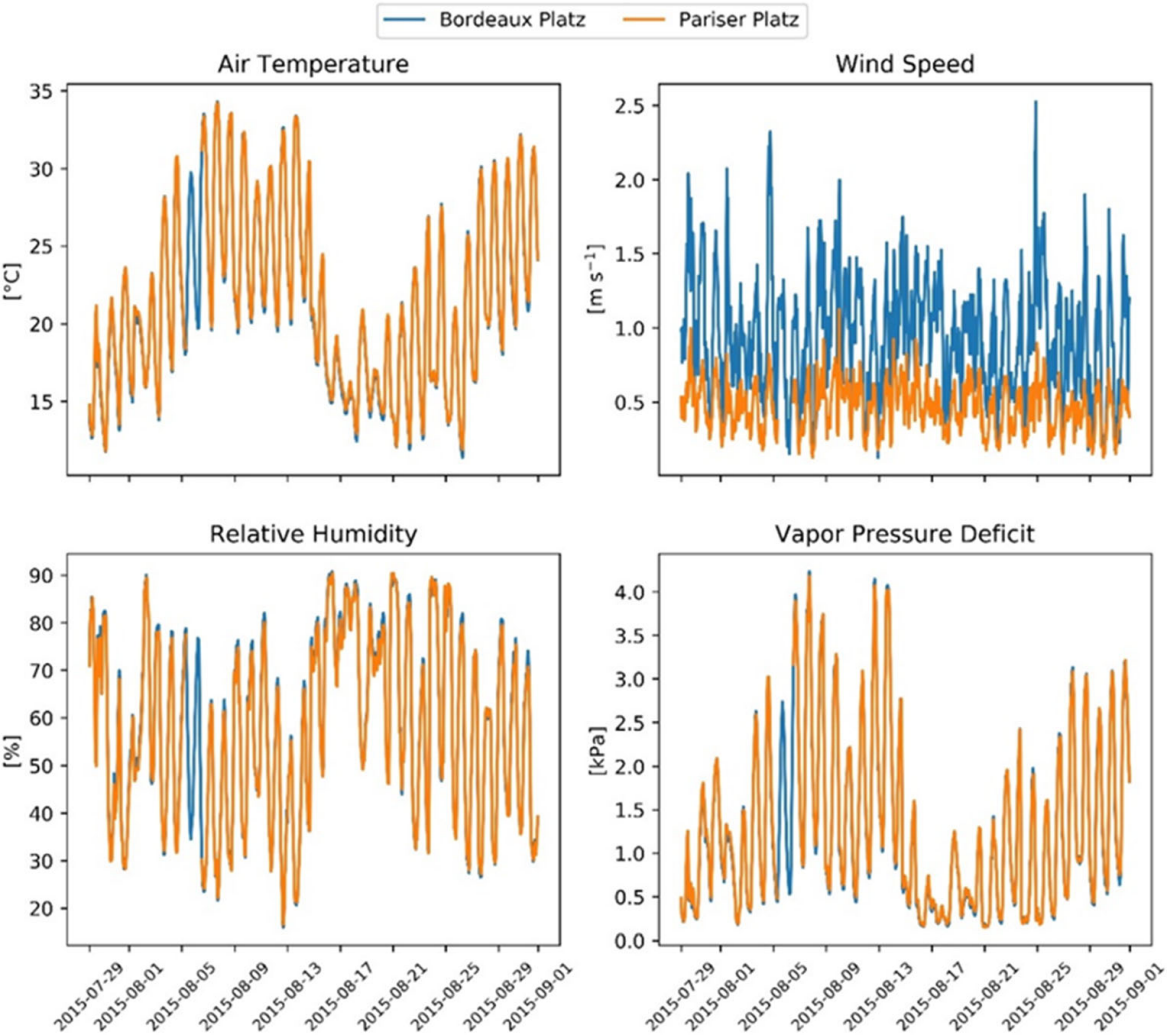
Meteorological conditions at the two study sites. Missing values in Pariser Platz on August 5–6, 2015

**Fig. 2:**
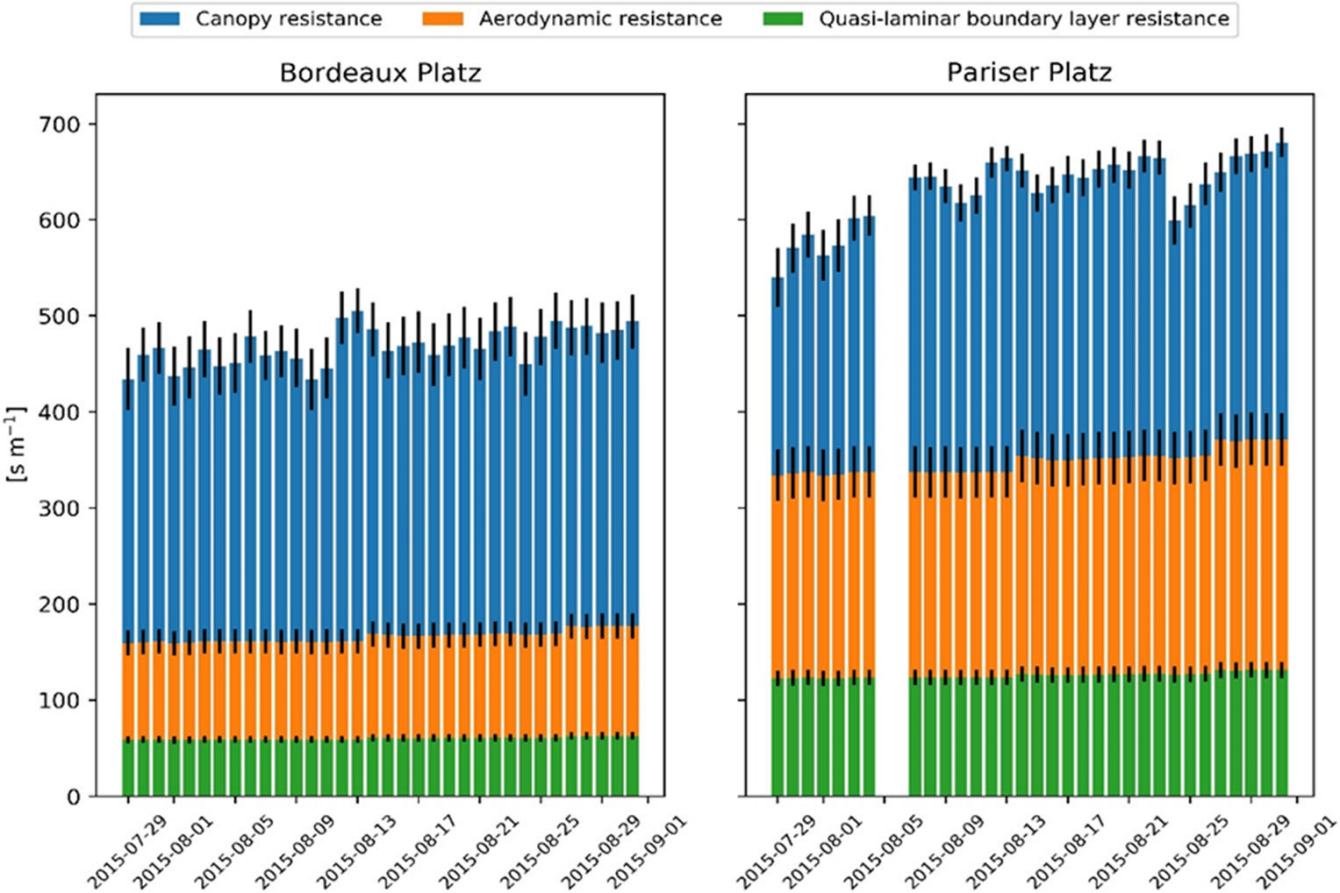
Model resistances at the two sites. The quasi-laminar boundary layer is referred to as O_3_

The two sites are not significantly different in terms of the air temperature, relative humidity, and vapor pressure deficit. However, wind speed at Bordeaux Platz (mean = 0.9 m s^−1^) is much higher than that at Pariser Platz (mean = 0.5 m s^−1^; Fig. 1). Consequently, the resistances of the aerodynamic and quasi-laminar boundary layer were considerably lower at Bordeaux Platz (on average 105.9 and 60 s m^−1^, respectively) than at Pariser Platz (222.4 and 125.9 s m^−1^, respectively; Fig. 2), which explains the higher transpiration demand at the former location.

**Fig. 3:**
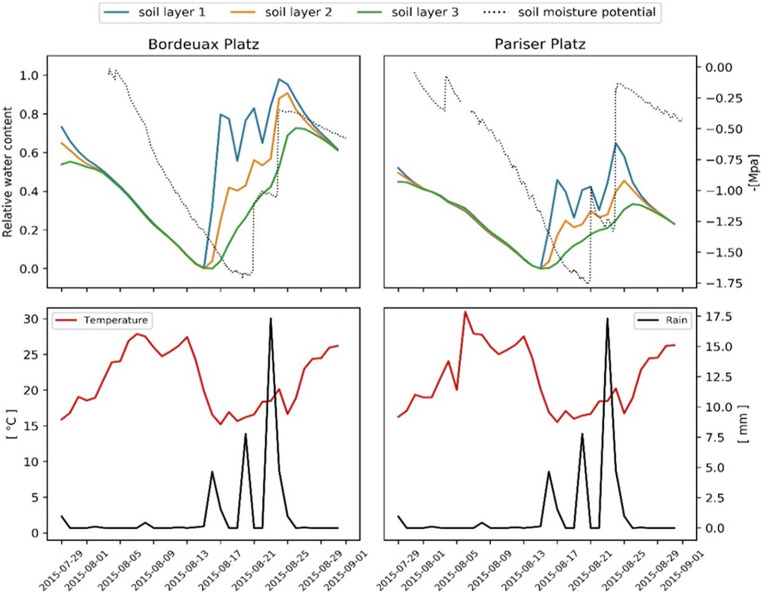
Upper panels: Relative water content in each soil layer and measured soil moisture potential. Lower panels: Average daily temperature and rain events in the study period

**Fig. 4:**
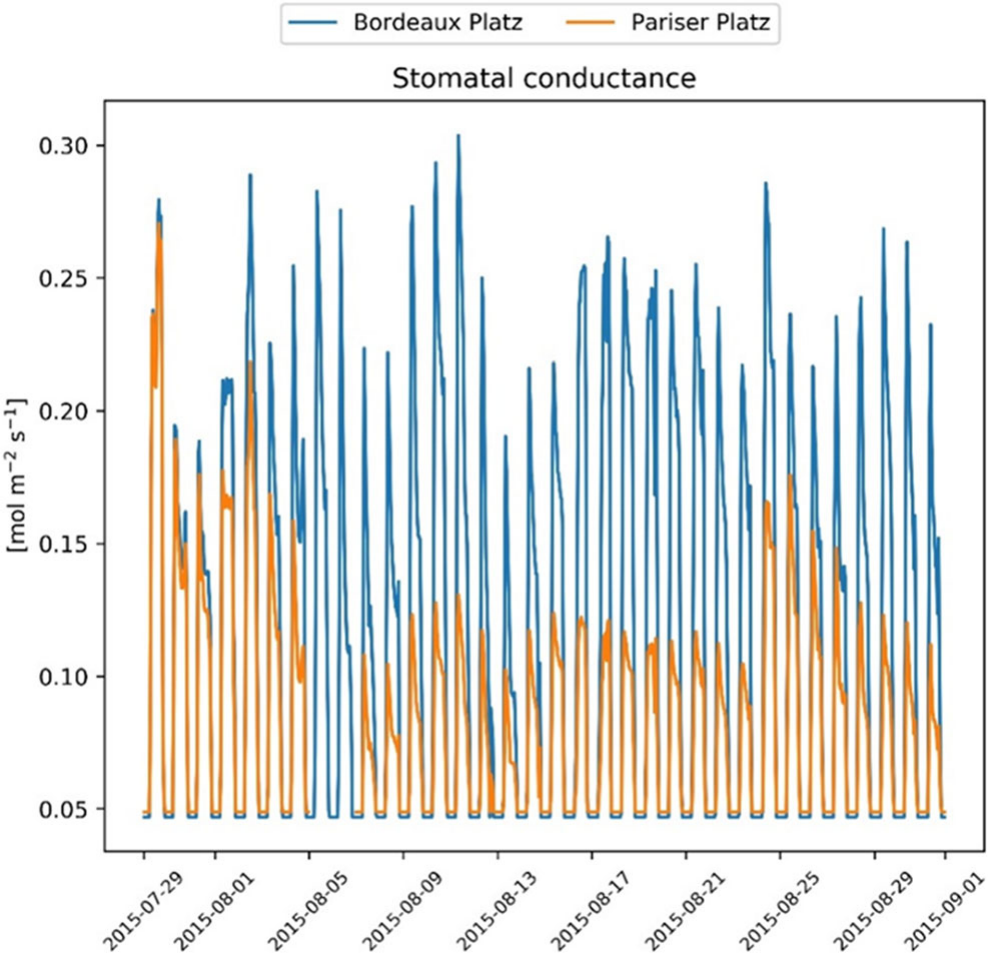
Stomatal conductance at the two sites

During the observation period, the negative soil water potential increased until mid-August when several precipitation events occurred, leading to the replenishment of the soil layers with water and a consequent increase in soil moisture potential. This trend could be represented with the simulation of soil water content, supporting the assumptions made about soil depth and surface sealing. However, recovery of the relative water content at Pariser Platz is lower than that at Bordeaux Platz (Fig. 3) because the water supply is reduced due to larger runoff. Therefore, drought at Pariser Platz is more prolonged, resulting in a persistently low stomatal conductance, while at Bordeaux Platz stomatal, conductance is increasing in the investigated period (Fig. 4).

**Fig. 5:**
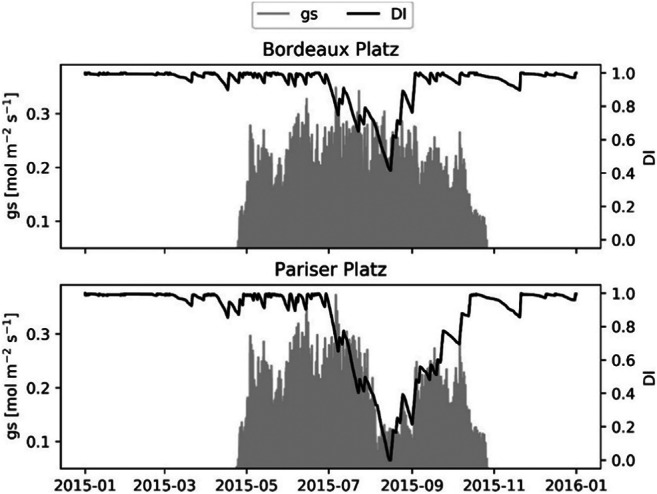
Drought index (DI) effect on stomatal conductance (gs) at the two sites calculated for the whole year 2015

In order to demonstrate the impact of drought, we simulated the development of the drought index (DI) on stomatal conductance throughout the whole year 2015 for both sites. At the Pariser Platz, the index drops almost to zero while it stays above 0.4 at Bordeaux Platz (Fig. 5). This considerable difference affects the Ball–Berry coefficient (*m*) and decreases stomatal conductance (gs) at the sealed sites relative to the open place.

**Fig. 6:**
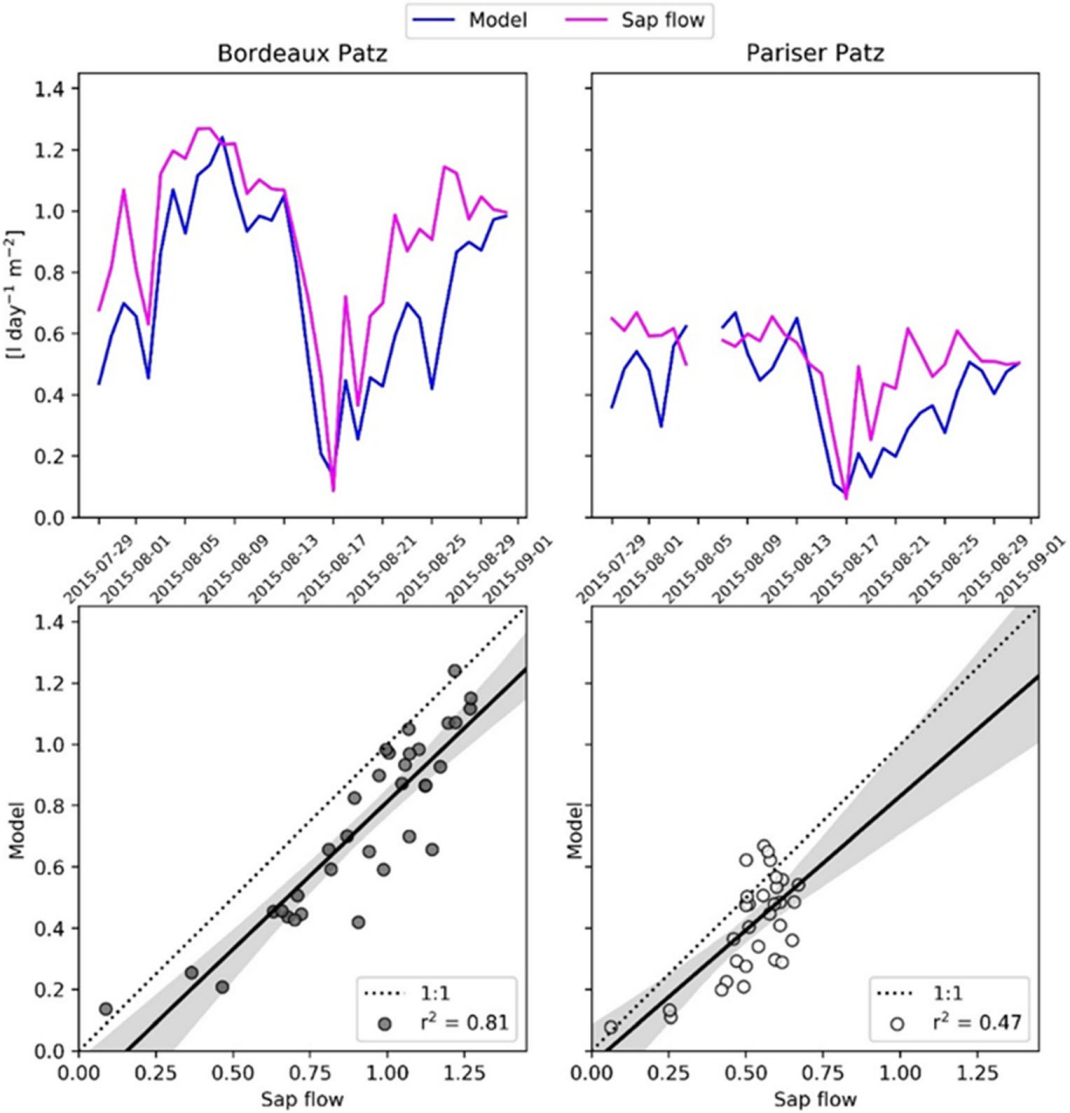
Top: Comparison of modeled transpiration and values measured using the sap flow for the sites Bordeaux Platz (left) and Pariser Platz (right). Bottom: Relationship between observed and simulated values

The modeled and measured transpiration values at the two sites overall agree, including the higher water losses at Bordeaux Platz due to higher stomatal conductance, highlighting the ability of the model to cope with the differences between the two sites (Fig. 6).

**Fig. 7:**
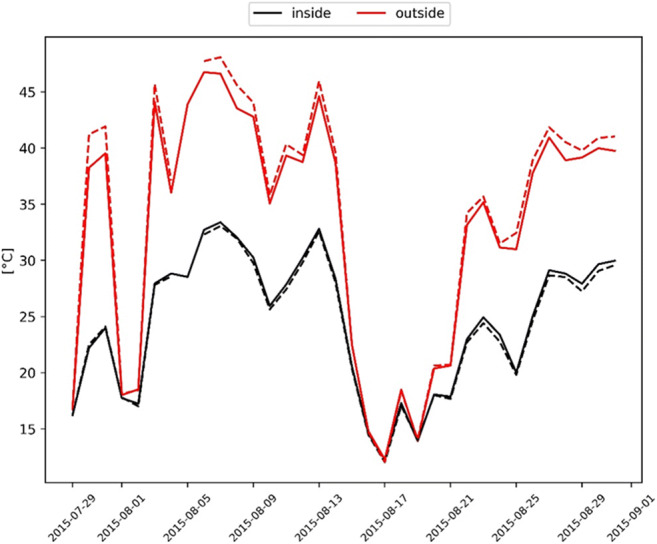
Simulated surface temperature (of an assumed concrete surface) outside and inside the tree canopy. The solid line is the simulation for Bordeaux Platz and the dashed line for Pariser Platz

In a model experiment, we can estimate the temperature effect originating from the shading of the crown to illustrate the usefulness of the model approach. Therefore, we calculate the temperature of an unshaded and a shaded concrete surface based on the recorded weather conditions at the two investigated places. This shows that the simulated surface temperature would be a lot lower under the tree canopy at the hottest days (up to 18.7 °C in Pariser Platz and 16.2 °C in Bordeaux Platz; Fig. 7), while during the cool period without much direct radiation, the shading effect is negligible. The differences in temperature regime between the places originate again predominantly from the lower wind speed at Pariser Platz, which limits the heat flow by convection to the surrounding air and thus leads to higher temperatures. On the other side, the surface temperature under shaded conditions at this site is slightly lower because of its slightly higher leaf area index.

**Fig. 8:**
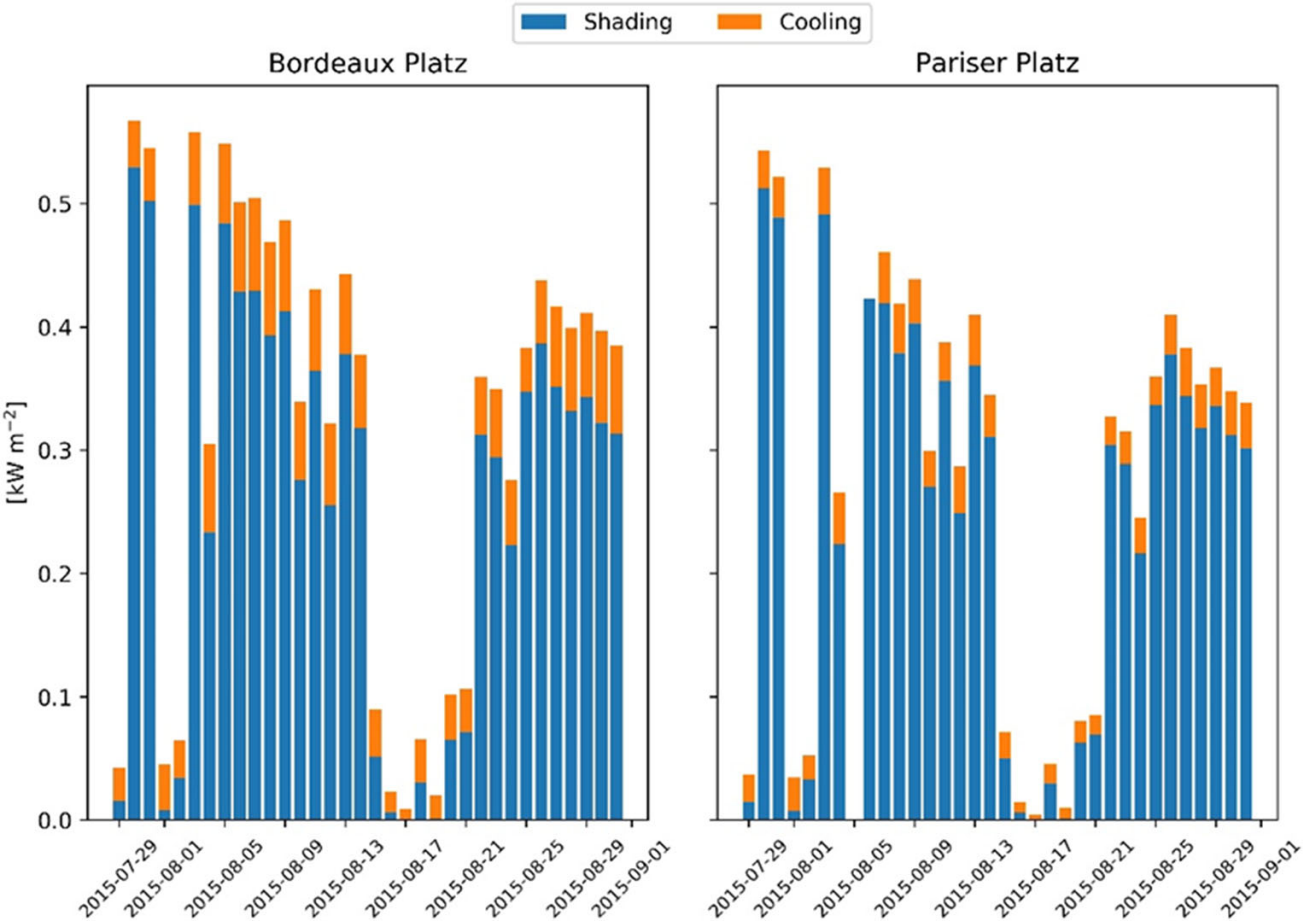
Energy reduction by concrete shading and cooling by transpiration at the two sites at midday

**Fig. 9:**
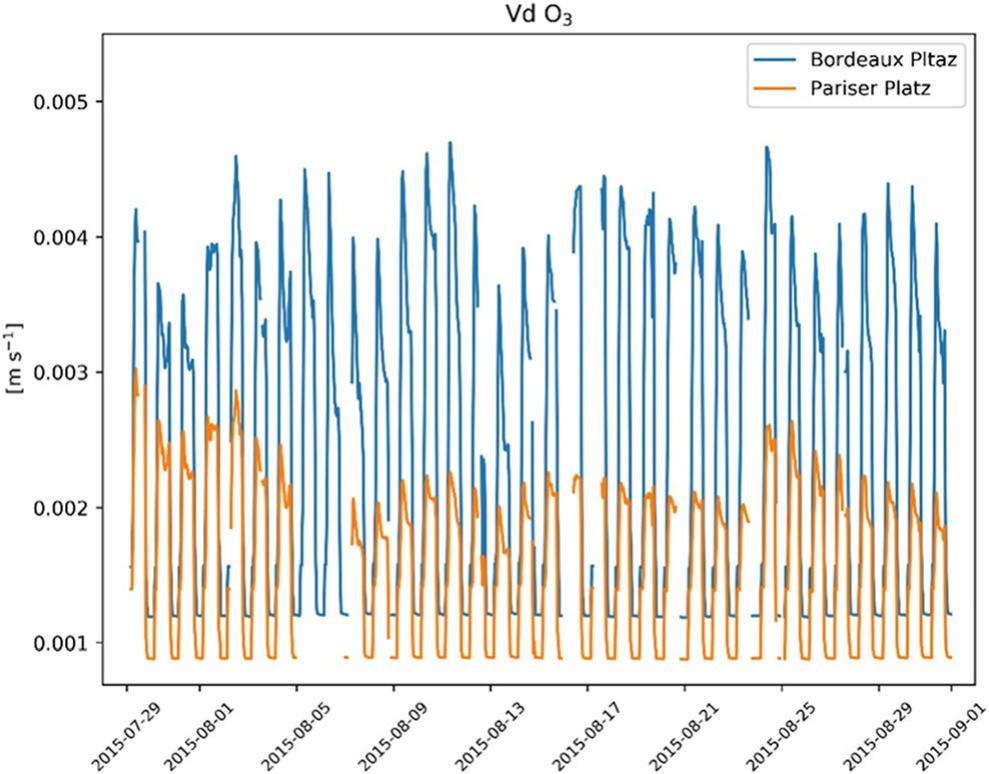
Simulated dry deposition velocity (vd) of O_3_ at the two sites. Missing values correspond to hours with precipitation. The differences of O_3_ uptake at the two sites result from the site-specific stomatal conductance and resistances

The transpiration performance particularly influences the cooling effect at Pariser Platz (mean = 0.03; max = 0.04 kW/m^2^) compared with Bordeaux Platz (mean = 0.05; max = 0.08 kW/m^2^). Instead, the energy reduction by shading is similar at the two locations (mean = 0.3; max = 0.5 kW/m^2^), indicating that the largest energy reduction occurs at midday (Fig. 8). The differences in the stomatal conductance result in remarkable differences in vd, as indicated in Fig. 9. Thus, it can be assumed that the conditions at Bordeaux Platz lead to a higher removal of gaseous pollutants than at Pariser Platz. For sites with comparable ozone formation and transport, this may ultimately lead to a relatively better air quality.

## Discussion

Urban trees can help mitigate urban extreme temperature events, which are expected to be more frequent and more severe in the future (Guerreiro et al. 2018). To better evaluate the efficiency of increased tree abundance, models are required that can be used to determine ecosystem services depending on both tree-specific properties and the (potentially changing) environment. Environmental conditions include direct influences of immediate climate and indirect influences, particularly the soil water availability (Livesley et al. 2016). Increased drought can be expected to affect the plant properties and thus the ecosystem services that are related to water evaporation (cooling) and pollution uptake (Stratópoulos et al. 2019; Zhang et al. 2020). Consequently, water availability impacts must be reflected in a model for decision support. Therefore, we implemented and tested a model that cannot only be used to directly calculate heat mitigation in terms of energy reduction due to cooling and shading but also considers the impact of drought stress on evaporation and the stomatal uptake. A similar approach that link different ecosystem services based on physiological responses has been very recently presented by Delaria and Cohen (2020) although not in the urban context.

The test simulations demonstrate that the energy reduction of urban trees is particularly high at midday on sunny and warm days due to the shading provided by tree crowns. The effectiveness of this process scales with LAI, crown depth, and width (Sanusi et al. 2017). Measurements of the pavement surface temperature for the study site in Munich were carried from Rahman et al. (2017b) reporting an average surface temperature reduction by 15.2 °C on three warm sunny days (July 21, August 08 and 13, 2015). Our simulations for the same days in August showed a similar average temperature reduction of about 13 °C for the two sites (Fig. 7). A recent study in which a thermal camera was used to determine the surface temperature change of asphalt, porphyry, and grass based on shading of different tree species, median average cooling values of 16.4, 12.9, and 8.5 °C was reported, respectively (Speak et al. 2020). In other studies, a maximum hourly cooling of 2.3 °C based on the shading of a single tree on the building facade (Zhao et al. 2018) and average cooling of the air temperature by trees in streets and courtyards of 2.5 °C (Shashua-Bar and Hoffman 2004) were determined.

The simulated transpiration effect depends on the meteorological conditions (wind speed, vapor pressure deficit) and soil water availability (pervious or impervious surfaces) and represents on average 12% of the total energy reduction (15% at Bordeaux Platz and 9% at Pariser Platz). Besides a higher aerodynamic and quasi-laminar boundary layer resistance, we attribute the result also to a lower soil water availability in Pariser Platz compared to Bordeaux Platz. It should be acknowledged that the simulated water content is not exactly in temporal agreement with measurements (Fig. 3). However, given the uncertainties in rainfall distribution and amount as well as soil property initialization, the results can still be seen as a confirmation of the model’s ability to represent drought. In particular, since it is corroborated by the direct transpiration measurements at both sites (Fig. 6).

The simulated transpiration is in overall well accordance with the measurements although recovery after cool periods is delayed by a few days. This is despite the simulated restriction by soil water limitation is less severe than indicated by measurements of soil water potential during the respective period. A possible explanation is that foliage and thus transpiration demand might increase faster in reality than assumed in the model which would call for a more comprehensive determination of surface temperature. Also, some differences are apparent between the development of soil water potential and the simulated soil water content, indicating either a very inhomogeneous precipitation distribution within the city or a non-linear relation between runoff and percolation that might need to consider ponding or channeling effects from gutters (Meili et al. 2020). The very scattered distribution of sensors, the scarce information about the soil properties, and the assumption of homogeneous soil sealing, however, demand for further evaluation studies in order to determine the importance of different foliage, surface, and soil properties to improve the respective parameterization.

Despite these uncertainties, the differences between the investigated sites have highlighted the importance of considering impervious surfaces, which characterize the urban fabric, enhances the water runoff, and reduces soil water infiltration (Wang et al. 2008). A limited water availability reduces the potential for evaporative cooling, in particular because of transpiration reduction (Gillner et al. 2015). This effect might significantly affect the immediate environment. For example, the results of experimental studies showed that evaporative cooling alone contributes to an air temperature reduction up to 3° within the canopies (Rahman et al. 2017b, 2020b) and the canopy-to-air temperature difference depends on meteorological conditions, tree species and, urban site-specific characteristics (Meier and Scherer 2012). The cooling effect by transpiration depends to a large extent on stomatal conductance (Tan et al. 2018) and our results are in agreement with measured values at midday on *Tilia europaea* in Sweden (0.1–0.2 mol m^−2^ s^−1^) (Konarska et al. 2016). The energy loss due to tree transpiration for commonly planted species in Central Europe including *T. cordata* ranges between 0.059 and 0.075 kW m^−2^ (Rötzer et al. 2019) which is similar to our results in Bordeaux Platz.

We demonstrate that the new model introduced in this study can capture the effect of drought on gaseous pollution uptake, as previously suggested by Wang et al. (2017). This may be of particular importance for future studies in Mediterranean cities characterized by climate with pronounced drought events; neglecting the effects of stomatal conductance may result in a significant overestimation of gaseous pollutant deposition (Morani et al. 2014). However, long periods of heat and drought exacerbated by the high percentage of sealed surfaces have also been recorded in cities at higher latitudes, which affected transpiration and reduced cooling (Gillner et al. 2015; Rahman et al. 2017a).

Note that only gaseous air pollution uptake was considered in this study because particle deposition is generally assumed to be independent of stomatal conductance. However, simple estimates based on the velocity of particulate matter that only depend on climate conditions (i.e., wind speed) and leaf area can easily be added (Pace and Grote 2020). Further improvements could then include, for example, that the stomatal conductance interacts with particle deposition, for example, the stomatal functionality decreases due to heavy particle absorption (Burkhardt and Pariyar 2014; Burkhardt et al. 2018). Nevertheless, we think that gaseous uptake by trees—not only of ozone but also of other oxidizing agents such as SO_2_, NO_2_, and NO—will increase in importance since these compounds are not only directly affecting human health but also increase allergenicity of pollen (Di Menno di Bucchianico et al. 2019). Considering species-specific properties is essential to maximize ecosystem services of urban trees because their features or performances are related to the climate and environmental conditions of the locations in which they grow (McCarthy et al. 2011). For example, under drought conditions, only species with anisohydric behavior will be able to provide cooling by transpiration as well as gaseous air pollution removal because other species will close their stomata early (Grote et al. 2016). Thus, it is important to describe both functions in dependence on stomatal conductance and also to determine conductance from water availability. The latter feature provides the means to define irrigation demand under the premise of a minimum conductance desired. Other examples that demonstrate the importance of considering species-specific differences when determining ecosystem services is that air pollution or/and high temperatures might particularly occur at times in the year when deciduous trees are leafless. In these circumstances, it is obviously desirable to plant evergreen trees, which have specific water uptake and deposition properties that a model need considering (Massetti et al. 2019). Overall, the arguments indicate that the features of the new model are important to determine ecosystem services of urban trees consistently, and we exemplarily demonstrated that the new model can be used for this purpose, i.e., quantifying temperature mitigation and pollution removal. In further applications, we will demonstrate that the model is also working on a larger range of environmental conditions as well as for different species based on the respective parameterization of crown properties, stomatal behavior, water-use efficiency, and photosynthesis (Kagotani et al. 2016; Stratópoulos et al. 2019).

We would like to highlight that several issues need to be considered in future research for further increases in the precision and usefulness of model-based assessment based on individual tree services. For example, the leaf area and related physiological processes depend on individual competition, which could be introduced to models to obtain a better individual performance estimate (Pace et al. 2018). On the process level, the interaction between ozone and the stomatal conductance might be introduced, that is, the decrease of the stomatal control under high ozone concentrations (Hoshika et al. 2018, 2020). The introduction of such a feature would also provide the means for considering the potential positive effects of reduced ozone concentrations on the water balances. In addition, seasonal responses of trees, such as leaf shedding, xylem embolism, and higher root turnover or the death of trees might be introduced in response to pollution or drought stress (Stratópoulos et al. 2019; Zhang et al. 2019). Finally, not only direct temperature effects but also changes in the air humidity should be simulated based on evaporation, which also improves the human thermal comfort and thus may add to the benefit of urban tree abundance (Upreti et al. 2017; Wang et al. 2018).

## Conclusions

Overall, it has been demonstrated that the newly introduced model can be used to calculate the temperature mitigation and pollutant deposition depending on environmental conditions and species-specific properties. The physiological basis facilitates the simultaneous and consistent consideration of direct shading by canopy coverage, cooling effects by transpiration, and uptake of gaseous pollutants. Based on the introduction of a simple water balance model, which is coupled to the stomatal conductance of trees, the effect of drought can be accounted for. The central role of stomatal conductance for cooling as well as air pollution removal also enables the consideration of pollution feedbacks on the physiology, although this remains to be implemented in the future. Already, the model might be applied for investigations covering longer time periods than one season and larger regions than just one site, but the evaluation for a wider range of environmental conditions (e.g., more prolonged droughts) remains to be demonstrated.
